
JOURNAL INFORMATION
==============================
NLM Title Abbreviation: Sci China Life Sci
Journal ID: Sci China Life Sci
NLM Journal ID: 101529880

Science China. Life Sciences

ISSN: 1674-7305
EISSN: 1869-1889

ARTICLE INFORMATION
==============================
PMCID: PMC7088800
PMID: 23737001
DOI: 10.1007/s11427-013-4496-y
Article ID: 4496
Article version: 1
Subjects: Commentary

Lessons learnt from the human infections of avian-origin influenza A H7N9 virus: Live free markets and human health

Wu Ying wuying@im.ac.cn
1
Gao George F gaofu@chinacdc.cn
1 2
1 grid.9227.e 0000000119573309 CAS Key Laboratory of Pathogenic Microbiology and Immunology, Institute of Microbiology, Chinese Academy of Sciences, Beijing, 100101 China
2 grid.198530.6 0000000088032373 Chinese Center for Disease Control and Prevention, Beijing, 102206 China
Electronic publication date: 2013 Jun 5
Print publication date: 2013
Volume: 56
Issue: 6
First page: 493
Last page: 494
Received 2013 May 19; Accepted 2013 May 20
Copyright: © The Author(s) 2013
License: Open AccessThis article is distributed under the terms of the Creative Commons Attribution 2.0 International License (https://creativecommons.org/licenses/by/2.0), which permits unrestricted use, distribution, and reproduction in any medium, provided the original work is properly cited.
License URL: https://creativecommons.org/licenses/by/2.0/

Keywords: Influenza, Avian Influenza, H7N9 Virus, Avian Influenza Virus, H7N9 Outbreak
issue-copyright-statement: © Science in China Press and Springer-Verlag GmbH 2013

==============================

WU Ying[1] & George F GAO[1,2]

Recent outbreak of avian-origin influenza A (H7N9) virus in the Yangtze River Delta area, China, expanding to neighboring areas in a later stage, with 36 deaths of 130 cases (as of May 16th) reminds the world of the threat avian influenza poses to the human health. Unlike the highly pathogenic H5N1 virus, which kills the birds [1–3], H7N9 is avirulent to either domestic poultry or migratory birds, which makes the situation even worse as it is hard to identify the birds who carry the viruses. In this issue of SCIENCE CHINA Life Sciences, Li et al. [4] present solid evidence that the human infecting H7N9 viruses are directly from the live free markets, where some patients visited before falling ill. In accordance with a report in The Lancet [5], the work also shows the avian-signature sequences from the environment where chickens are kept in the free market and human-signature sequences in the patients, implying the *in-vivo* micro-evolution and host adaptation during the outbreak and infection.

When the newly-emerging H7N9 was first diagnosed [6], avian-origin was suspected but there was no clue as to the real origin of the virus as neither sick birds had been seen nor the virus had been detected from any birds (domestic or migratory). Earlier phylogenetic and coalescent analyses based on limited whole-genome sequences by our group [7] reveal that the virus is a 4-origin reassortment and the recent reassortment event occurs in the Yangtze River Delta, with H7 and N9 gene segments coming from, possibly migratory birds, and all the other 6 internal genes from local chickens in the H9N2 virus background. Again, this event addresses the significant roles the migratory birds play in the influenza virus reassortments. Then some clues emerge indicating the local domestic poultry, esp. chickens, might carry the reassorted viruses, even the reassortments might occur in the domestic chickens. Later the viruses were isolated from pigeons, quails and their environments [8]. Now Li et al. [4] report three H7N9-infected patients and one of them visited a live free market. Virus genome sequence from this particular patient matched well with the virus sequence derived from the swab of the chicken cage in the market, except for the chicken- versus human-signature substitutions. The direct linkage of the chicken materials with the patient has been established. This shows the close-down of the live free market indeed has played a critical role in effectively controlling this H7N9 outbreak. Preliminary epidemiology data show that roughly about 75% patients could trace back with possible poultry (or poultry market) contacts. There might be common exposure to the poultry environment even in a few family cluster infections. Therefore direct contacts with live poultry or poultry markets are the source of the infection.

Based on these findings, the health authorities in the Yangtze River Delta areas immediately introduced the live market close-down measures and eventually the infected cases have gone down. With this outbreak we learnt that the traditional farming system and live free market indeed have some impacts on the emergence of new pathogens. There are some discussions as to whether or not we should permanently shut down the live market as it challenges the traditional life style and has side-effect on the economy. Agriculture sector argues that the H7N9 does not cause any diseases for poultry therefore it should not be called avian influenza virus. Whereas Li et al. [4] unambiguously demonstrated the relatedness of human infection with poultry or poultry related market. The question can be open for further discussion and we also need to see the circulation trends of the virus in the future. At least this virus should be called avian-origin influenza virus. Preliminary estimates show that the agriculture sector lost at least 57 billion RMB because of the live free market shut-down. Therefore this outbreak is not only a health problem but also a serious economic issue. Burden of disease (BOD) caused by this H7N9 outbreak should be accessed in the near future to guide our future disease control and prevention.

It has been estimated that 75% of newly emerging human-infecting pathogens are animal-borne or animal-origin. Due to ecological changes and intervention of animal habitat with human activities more and more zoonotic diseases strike human beings. While we are tackling with the H7N9 challenges, more cases of the new coronavirus, 2012-emerged novel human coronavirus Erasmus Medical Center (hCoV-EMC), have been confirmed in the Middle East, France and Germany (www.who.org); the virus expands its border. Last year, HIV positive individuals increased a significant number in China. Therefore we are facing ecological and behavioral infectious diseases in modern society, we ought to think hard and get prepared while enjoying ourselves with swift economic development. As a matter of fact, eco-health concept has been proposed for a while. One world, one health, to control the emerging pathogens we need to have a harmonious relationship with not only ourselves, the human beings, but also with our environment and the animals on the earth. We need to also change, somehow, our life styles, if necessary, like the live free market.

The emergence of H7N9 virus reminds the world of the threat with any unexpected pathogens. Since the first human infection of H5N1 avian influenza virus was diagnosed in Hong Kong in 1997, we have been talking about H5N1, H9N2, H7N3, H7N7, etc. causing human infections, but never even thought about H7N9. This H7N9 virus is the first neuraminidase (NA) subtype N9 seen to cause human infection. This reminds us that everything is possible. We should stay open-minded to carry out an extensive surveillance of influenza viruses for a better pre-warning system to be established globally.

**Open Access** This article is distributed under the terms of the Creative Commons Attribution License which permits any use, distribution, and reproduction in any medium, provided the original author(s) and source are credited.

This article is published with open access at Springerlink.com


References

1 Gao G F Shaw P C The challenges of avian influenza virus: mechanism, epidemiology and control Sci China Ser-C Life Sci 2009 52 405 406 10.1007/s11427-009-0058-8 PMC7089393 19471861
2 Liu D Liu X L Yan J H Interspecies transmission and host restriction of avian H5N1 influenza virus Sci China Ser-C Life Sci 2009 52 428 438 10.1007/s11427-009-0062-z PMC7089370 19471865
3 Chen H L H5N1 avian influenza virus in China Sci China Ser-C Life Sci 2009 52 419 427 10.1007/s11427-009-0068-6 19471864
4 Li J Yu X F Pu X Y Environmental connections of novel avian-origin H7N9 influenza virus infection and virus adaptation to the human Sci China Life Sci 2013 56 493 494 10.1007/s11427-013-4496-y 23657795
5 Chen Y Liang W Yang S Human infections with the emerging avian influenza A H7N9 virus from wet market poultry: clinical analysis and characterisation of viral genome Lancet 2013 368 1888 1897 10.1016/S0140-6736(13)60903-4 PMC7134567 23623390
6 Gao R Cao B Hu Y Human infection with a novel avian-origin influenza A (H7N9) virus N Engl J Med 2013 10.1056/NEJMoa1304459 23577628
7 Liu D Shi W Shi Y Origin and diversity of novel H7N9 avian influenza viruses causing human infection Lancet 2013 10.1016/S0140-6736(13)60938-1 23643111
8 Shi J Z Deng G H Liu P H Isolation and characterization of H7N9 viruses from live poultry markets—Implication of the source of current H7N9 infection in humans Chi Sci Bull 2013 58 1857 1863 10.1007/s11434-013-5873-4
